# Clinical Features and PTCH1 Expression in Gorlin–Goltz Syndrome: A Case Report †

**DOI:** 10.3390/reports8010034

**Published:** 2025-03-18

**Authors:** Gabriela González-López, Samuel Mendoza-Álvarez, Claudia Patricia Mejia-Velazquez, Carla Monserrat Ramírez-Martínez, Alejandro Alonso-Moctezuma, Luis Fernando Jacinto-Alemán

**Affiliations:** 1 Department of Oral Pathology and Medicine, Postgraduate Division, School of Dentistry, National Autonomous University of Mexico, Mexico City 04510, Mexico; gabrielagonzalezlo@comunidad.unam.mx (G.G.-L.); coor.pato-depei@fo.odonto.unam.mx (C.P.M.-V.); c.ramirez@fo.odonto.unam.mx (C.M.R.-M.); 2 Oral and Maxillofacial Surgery Specialty, Postgraduate Division, School of Dentistry, National Autonomous University of Mexico, Mexico City 04510, Mexico; samuel.mendoza.alvarez@gmail.com (S.M.-Á.); alonsomoctezuma@fo.odonto.unam.mx (A.A.-M.)

**Keywords:** Gorlin–Goltz syndrome, odontogenic keratocysts, PTCH1, RT-PCR, basal cell carcinoma

## Abstract

**Background and Clinical Significance:** Basal cell nevoid carcinoma syndrome, or Gorlin–Goltz Syndrome (GGS), is a genetic disease caused by germline mutations in genes involved in the Sonic HedgeHog (SHH) signaling pathway, mainly in the PTCH1 gene. PTCH1 is a receptor for SHH, and the activation of SHH signaling exerts a direct effect on the proliferation and maintenance of stem cells; alteration of its signaling could promote a favorable microenvironment for the maintenance of tumor viability. The main clinical manifestations of patients with GGS include multiple basal cell carcinomas, odontogenic keratocysts, calcification of the falx cerebri, palmoplantar fossae, hypertelorism, prognathism, fused or bifid ribs, and macrocephaly, which occur at different stages of life. **Case Presentation**: Here, the case of a 48-year-old woman is described, for whom a clinical and histopathological diagnosis of GGS was made due to the presence of two major criteria (multiple odontogenic keratocysts and calcification of the falx cerebri) and one minor criterion (congenital anomalies), according to Kimonis. Additionally, an end-point RT-PCR assay showed a decrease in PTCH1 gene expression. A conservative therapy was established, and satisfactory results were obtained in a follow-up period of 18 months. **Conclusions**: Kimonis' clinical criteria are important for establishing the diagnosis of Gorlin syndrome.

## 1. Introduction and Clinical Significance

Basal cell nevus carcinoma syndrome, also known as Gorlin–Goltz syndrome (GGS), was first described in 1960 by Gorlin and Goltz as a complex multi-organ autosomal dominant entity caused by germline variants in genes involved in the Sonic HedgeHog (SHH) signaling pathway, mainly the PTCH1 (Patched 1) gene in approximately 40–80% of cases. This gene is located on chromosome 9q22.3. The estimated prevalence of GGS varies from 1/19,000 to 1/164,000 cases, depending on the population studied [1,2,3].

The SHH signaling pathway is essential for proliferation and cell differentiation, as well as the regulation of embryogenesis; however, loss of PTCH1 function can trigger an uncontrolled increase in the activity of this pathway [4]. PTCH1 is the receptor for the SHH ligand. In the absence of the SHH ligand, PTCH1 suppresses SMO activity and blocks the expression of transcription factors of the GLI family. Mutations that cause the functional loss of PTCH1 allow for the activation of transcription factors that promote cell growth. SHH signaling exerts a direct effect on the proliferation and maintenance of stem cells, and their alteration in stroma paracrine signaling could promote a favorable microenvironment for tumor viability [5].

GGS leads to skeletal, stomatognathic, and central nervous system alterations, among others. Examples of such head and neck abnormalities include odontogenic keratocysts, ectopia, dental agenesis, calcification of the falx cerebri, congenital hydrocephalus, pons sella turcica, cataracts, coloboma, microphthalmia, and strabismus. Additionally, patients have an increased risk of developing tumors such as medulloblastoma or ovarian and cardiac fibromas [6].

In 1997, Kimonis et al. proposed a list of clinical criteria for the diagnosis of GGS. Identification of two major criteria, one major and two minor criteria, or one major criterion as well as molecular confirmation are required to establish the diagnosis (Table 1) [7,8,9].

In the head and neck region, the most frequent clinical–pathological finding is odontogenic keratocysts (OKCs). The overall prevalence of these lesions is 66–86%; however, the time of presentation is age-dependent, at around 40 years of age [1].

OKCs are characterized by aggressive local growth and a high recurrence rate [10,11]. They are most frequently located in the posterior mandibular region with asymptomatic anteroposterior growth that causes cortical expansion. Their post-treatment recurrence rates range from 60% after curettage and enucleation to 20% when they are treated via peripheral ostectomy or cryotherapy, and close to 0% after bloc resection [3]. Histologically, they are characterized by a lining of stratified parakeratinized squamous epithelium, whose basal cells present nuclear hyperchromasia and are arranged in a palisade [1].

Therefore, our aim was to present a case report of GGS diagnosed in our service, according to Kimonis et al [7]., along with the associated molecular findings.

## 2. Case Presentation

**Figure 1:**
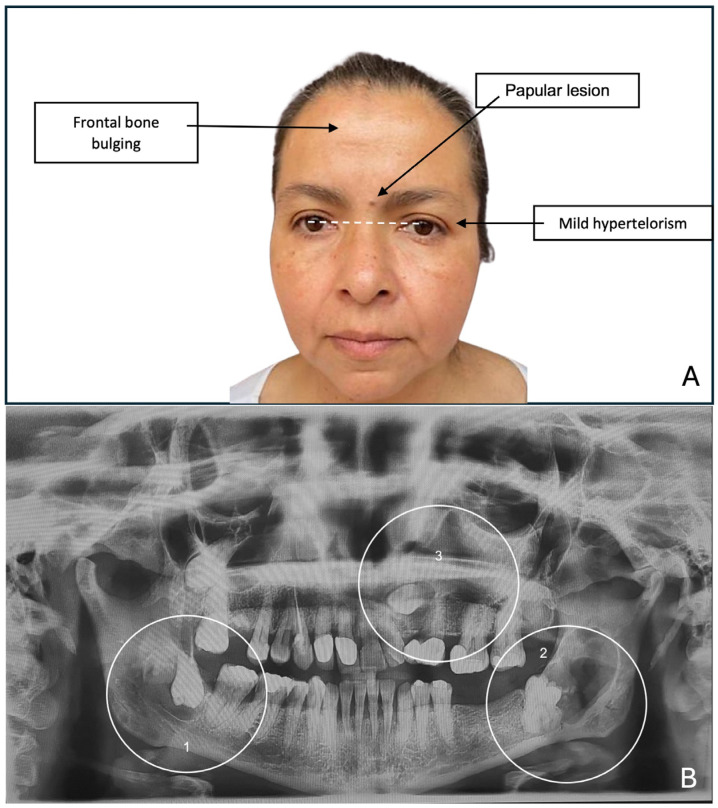
(**A**) Extraoral photograph of the patient showing thick facies, frontal bone bulging, mild hypertelorism, and a lesion on the skin of the interciliary region. (**B**) Orthopantomography showing all lesions: 1—mandibular lesion on the right side; 2—mandibular lesion on the left side; 3—lesion involving the maxillary sinus on the left side.

A 48-year-old woman came to the clinic due to the discovery of multiple radiolucent lesions in both jaws, identified during an imaging evaluation for comprehensive rehabilitation treatment in the Postgraduate and Research Division of the Dental School, UNAM. Upon examination, she reported a history of solid basal cell carcinoma on the skin of the lower eyelid at 44 years of age. During the extraoral exploration, thick facies with frontal bone bulging, mild hypertelorism, short neck, and low insertion of the auricles were identified; in addition, an asymptomatic papular 0.3 cm diameter lesion was found located on the interciliary skin region with a smooth surface, light brown color, firm consistency, and unknown evolution time (Figure 1A). In the malar region and nasal bridge, small melanocytic macules of approximately 5 mm were observed. The orthopantomography findings showed multiple radiolucent areas; the first lesion was located in the right ascending mandible ramus with an approximate diameter of 2 cm, showing well-defined sclerotic edges surrounding the crown of the third molar, impacting and inverting it. The second lesion was in the ascending left ramus of the mandible, measuring 2.5 cm in diameter, with well-defined edges, distal to a partially erupted third molar, and the third lesion was located in the left maxillary sinus zone, with a diameter of 2 cm and well-defined edges involving the impacted canine (Figure 1B).

**Figure 2:**
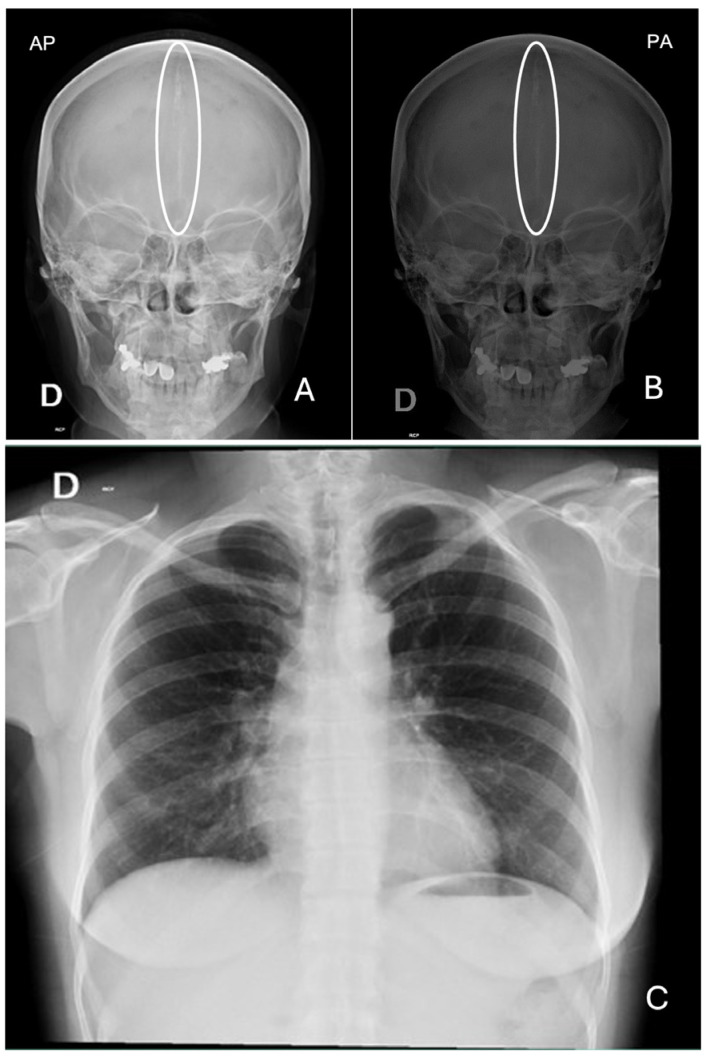
(**A**) Anteroposterior and (**B**) posteroanterior projection of skull X-ray showing a radiopaque area in the midline corresponding to falx cerebri calcification. (**C**) Chest X-ray, in which no alterations were identified.

The initial differential diagnoses included dentigerous cysts versus odontogenic keratocysts. However, considering the multiplicity of the lesions and the patient’s facial characteristics, GGS was suspected; thus, complementary studies of chest and skull X-rays were requested. The findings were as follows: skull X-ray in anteroposterior projection showing the presence of calcification in the topography of the falx cerebri, and chest X-ray showing normal radiographic parameters (Figure 2).

**Figure 3:**
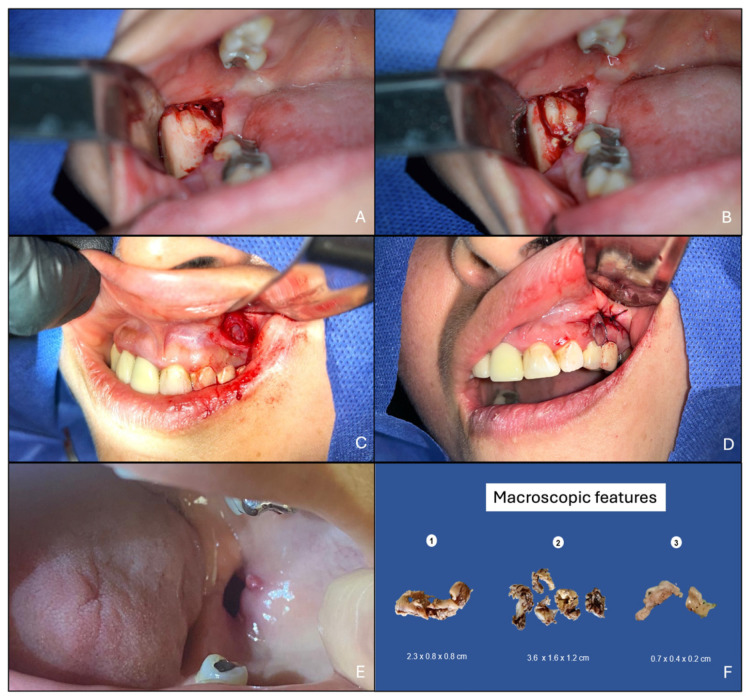
Surgical approach for excisional biopsy and macroscopic features. (**A**) Surgical approach to the mandibular right lesion, (**B**) discharge of dirty white liquid common in OKCs, (**C**) surgical approach to the maxillary lesion, (**D**) drain placement for decompression, (**E**) residual wound after 5 months, and (**F**) macroscopic lesion aspects: (1) lesion of the right mandibular ramus, (2) lesion of the left mandibular ramus, and (3) lesion of the left maxillary sinus.

First, an incisional biopsy was performed to corroborate the diagnosis; at the same time, a drain was placed inside the cavities and fixed with suture and wire to perform cyst decompression, and the patient received instructions to wash the cavities three times a day with pure water. The drain was left for 5 months to perform total excision of the lesions posteriorly, with additional local curettage and placement of 5% fluorouracil; enough 5% fluorouracil was applied to moisten a gauze that was placed inside the cavity, which was removed 24 h later. The wounds were closed due to the smaller size (Figure 3A). Macroscopic analysis of the enucleated specimens indicated that all had a capsular appearance, light brown color, rubbery consistency, and uneven surfaces (Figure 3B).

**Figure 4:**
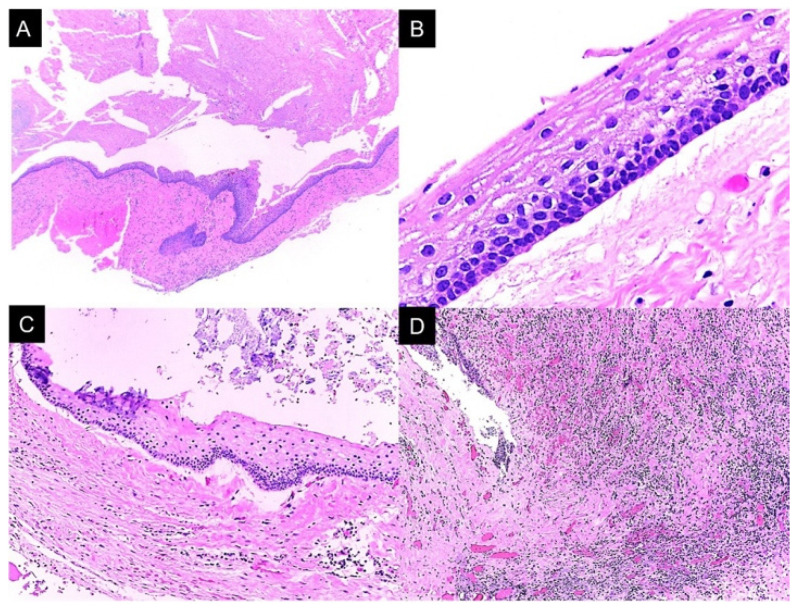
Lesion 1 histopathological features. (**A**) Cystic lesion with abundant keratin content and an island of odontogenic epithelium in its wall. (**B**) Parakeratinized stratified squamous epithelium lining with palisade-shaped basal cells and nuclear hyperchromasia. (**C**) Separation between the epithelial lining and the lesion wall. (**D**) Severe and diffuse lymphoplasmacytic inflammatory infiltrate. Photomicrograph (**A**) at 40×, photomicrograph (**B**) at 400×, and photomicrographs (**C**,**D**) at 100×.

The histopathological analysis of lesion 1 (right mandibular ramus) showed a cystic lesion lined with parakeratinized stratified squamous epithelium, nuclear hyperchromasia in basal cells, and a palisade arrangement, with an island of inactive odontogenic epithelium and severe lymphoplasmacytic inflammatory infiltrate. In the cystic lumen, the lesion, keratin remains, erythrocyte extravasation, and fissures formed by cholesterol crystals were observed. The epithelium–connective tissue interface was flat, with epithelial lining detachment (Figure 4).

**Figure 5:**
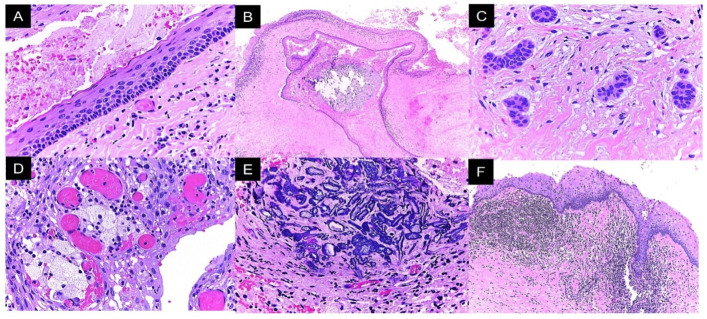
Lesion 2 histopathologic features. (**A**) Cystic lesion lined by epithelium characteristic of an odontogenic keratocyst, (**B**) presence of a satellite lesion in the wall of the lesion, (**C**) inactive odontogenic epithelium remnants, (**D**) mucous cell metaplasia in the epithelial lining, (**E**) calcified matrix deposits, and (**F**) abundant lymphoplasmacytic inflammatory infiltrates in the cyst wall with foci of acanthosis and spongiosis in the epithelium. Photomicrographs (**A**,**C**–**E**) at 400× and photomicrographs (**B**,**F**) at 100×.

Lesion 2 (left mandibular ramus) was a cystic lesion lined with parakeratinized stratified squamous epithelium, with corrugated surfaces and uniform thickness. The basal cells showed a cubic morphology and palisade arrangement with hyperchromatic nuclei. Mucosal metaplasia, satellite lesions, and odontogenic epithelium cords with abundant chronic inflammatory infiltrate and areas of calcification were observed in the wall. Abundant cellular debris and erythrocyte extravasation were identified in the cystic lumen (Figure 5).

**Figure 6:**
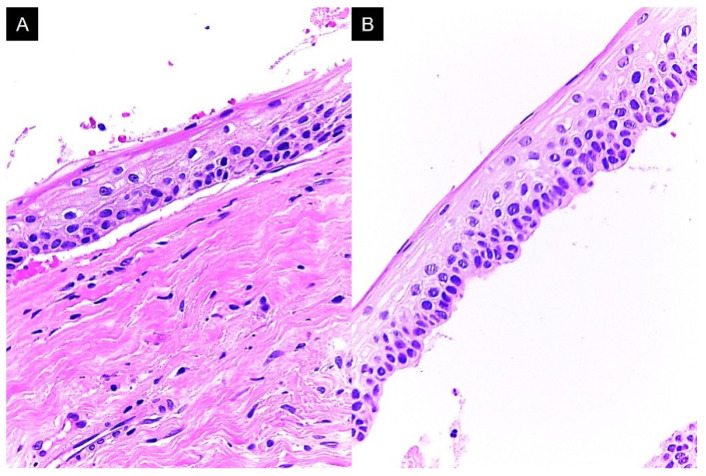
Lesion 3 histopathological features. (**A**) Cystic lesion lined by epithelium characteristic of odontogenic keratocysts; (**B**) complete detachment of the epithelial lining. Both photomicrographs are at 400×.

In lesion 3 (left maxilla), we observed a cystic stratified parakeratinized squamous epithelium with uniform thickness, basal cells organized in a palisade arrangement, and nuclear hyperchromasia. In some areas, epithelial lining and connective wall detachment was observed (Figure 6). The histopathological diagnosis for the three lesions was odontogenic keratocysts.

**Figure 7:**
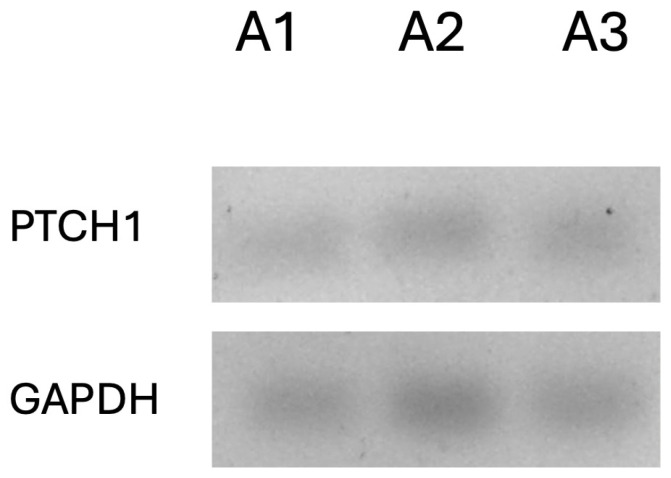
PTCH1 densitometric analysis expression (triplicate RT-PCR). PTCH1 showed lower density than GAPDH (control gene). A1: Assay 1; A2: Assay 2; A3: Assay 3.

The end-point RT-PCR assay was used to determine the expression of the PTCH1 gene. First, 150 µm of three lesions (50 µm each) was obtained to perform total RNA extraction using the commercial AllPrep DNA/RNA FFPE Kit (80234, Qiagen, Hilden, Germany). The quality and quantity of total RNA were determined using a NanoDrop ND-2000 spectrophotometer (Thermo Fisher, Rochester, NY, USA); then, we performed PTCH1 gene (NM_000264.3) amplification using the Access RT-PCR system (Promega, Madison, WI, USA) with its specific probes (sense: GGGTGGCACAGTCAAGAACAG; antisense: CGTACATTTGCTTGGGAGTCATT), as reported [12], resolving the products in a 3% agarose gel stained with ethidium bromide for visualization in an Axygen photodocumentation system (Axygen, Tewksbury, MA, USA). Optical density normalization was performed using the GAPDH gene (sense: AGGGCCCTGACAACTCTTTT; antisense: AGGGGTCTACATGGCAACTG) as a constitutive control; the assay was performed in triplicate. The PTCH expression was 44% lower than the basal control expression (Figure 7).

**Figure 8:**
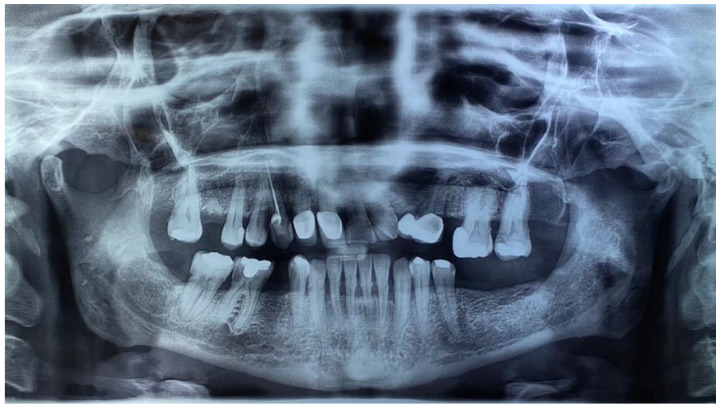
Control orthopantomography at 18 months after surgical intervention.

In accordance with the guidelines of Kimonis et al [7]., this case satisfied two major criteria and one minor criterion, which was sufficient to establish a diagnosis of Gorlin–Goltz syndrome. When informing the patient of the diagnosis, we asked whether her parents or any relatives presented similar symptoms; the patient denied the possibility. Eighteen months after the surgical intervention, an orthopantomography control showed bone apposition without new lesions (Figure 8).

## 3. Discussion

The diagnosis of GGS is based on clinical manifestations; however, the phenotype is variable, as there are patients who present many characteristics and other patients who show minimal data, meaning the diagnostic challenge requires complementary tests [13]. In the present case, two major criteria (odontogenic keratocysts and calcification of the falx cerebri) plus one minor criterion (congenital anomaly corresponding to frontal bone bulging and hypertelorism) were identified. Although the patient reported enucleation of basal cell carcinoma (BCC) in the lower eyelid, and in the clinical examination, there was a suggestive lesion in the interciliary region—which could be another BCC—we did not have histopathological confirmation; therefore, we only considered the confirmed findings. Patients with GGS are very sensitive to radiation and tend to develop basal cell carcinomas in the radioexposed field [9]. BCC is a tumor that rarely metastasizes; however, it has been associated with significant morbidity due to its potential for local invasion that promotes tissue destruction [5].

OKCs are usually the first dental manifestation detected via dentistry. They are commonly detected as incidental findings during normal or orthodontic consultation [14,15]. Our patient showed three lesions (two in the mandibula and one in the maxilla), which pointed to a syndromic condition. It has been described that syndromic OKCs present histopathological differences to sporadic ones, such as epithelium budding and mitoses, an abundance of satellite lesions, and solid islands of odontogenic epithelium in the capsule [16,17,18]. Our case contained these two latter features. It has been reported that these findings could be related to modifications in molecular cystic pathogenesis.

PTCH1 is a gene located on the 9q22.32 chromosome, which codes for a receptor for SHH. It is considered a tumor suppressor gene because its inactivation could be a risk factor for tumorigenesis [19]. Nearly 90% of BCCs present inactivation of at least one PTCH1 allele and mutations in the activation of SMO in 10–20% of cases [5,9].

Evans et al. reported that PTCH1 is altered in 90% of patients with OKCs and macrocephaly [20]. Our end-point RT-PCR analysis suggested a reduction in the expression of PTCH1. This finding could be associated with a possible mutation or alteration in the transcription process, which is manifested as a reduced expression. To confirm this finding, it is necessary to perform gene sequencing and determine the molecular alteration responsible for this expression decrease [21].

The current treatment for keratocysts is divided into (1) conservative treatment, (2) radical or aggressive treatment, and (3) adjuvant treatments. The conservative methods include simple enucleation with primary closure, decompression, or marsupialization; cryosurgical techniques (with liquid nitrogen); chemical destruction using 5-fluorouracil and application of Carnoy’s solution (previously consisting of a mixture of absolute alcohol, chloroform, glacial acetic acid, and ferric chloride; chloroform is no longer used due to its carcinogenic potential) or, currently, Modified Carnoy’s solution; and radical surgical techniques with bone resection [22].

Conservative surgical treatments such as decompression are chosen in young patients due to subsequent epithelium metaplasia changes and the thickening of the cystic wall, which facilitate enucleation [17,23].

A conservative therapeutic approach was employed for this patient with additional 5% fluorouracil application, obtaining favorable results. Fluorouracil at 5% is an antimetabolite that attacks DNA synthesis in proliferating cells via the specific inhibition of thymidylate synthase, causing cellular apoptosis, and has been widely used as a chemotherapeutic agent for various neoplasias and OKCs [24,25]. As an adjuvant, 5-fluorouracil has shown promising results with OKC. It is a fluorinated analog of uracil that induces cell apoptosis through downregulation of the Hedgehog (HH) signaling pathway [3,22,26]. After enucleation and peripheral osteotomy of the OKC, it was covered with sterile gauze with 5% 5-FU and packed into the surgical wound. The wound was closed in the usual manner, leaving a small distal end piece (approximately 1 cm) of gauze exposed to allow the gauze to be removed at 24 h post-operatively. Its use resulted in normal bone healing without local or systemic adverse effects [23]. Recent studies show that the recurrence rate of 5-fluorouracil after peripheral enucleation and osteotomy is 0% [23].

## 4. Conclusions

GGS is a complex multi-organ disease that requires interdisciplinary management to treat lesions with minimal or no complications. Therefore, close patient monitoring to identify new lesion development, such as OKCs or BCC, is important.

Most cases are diagnosed during routine evaluation in dental offices; therefore, dentistry could be the first contact for GGS diagnosis when multiple cystic lesions are observed in an orthopantomography examination. Eighteen months after surgery, no lesion was identified; however, genetic counseling for the patient and family members is necessary.

## Figures and Tables

**Table 1:** Diagnostic criteria for Gorlin–Goltz syndrome proposed by Kimonis.

Major Criteria	Minor Criteria
More than two basal cell carcinomas or one in a patient under 30 years of age	Macrocephaly determined after size adjustment
Histologically confirmed odontogenic keratocysts	Congenital malformations: cleft lip or palate, bulging of the frontal bone, coarse facies, hypertelorism
Three or more palmoplantar pits	Skeletal abnormalities: Sprengel deformity, syndactyly
Bilamellar calcification of the falx cerebri	Radiological abnormalities: pons sella turcica, vertebral abnormalities such as fusion or elongation of the vertebral bodies
Bifid or fused ribs	Ovarian fibromas
First-degree relative with GGS	Medulloblastoma

## Data Availability

The original contributions presented in this study are included in the article. Further inquiries can be directed to the corresponding author.

## Abbreviations

The following abbreviations are used in this manuscript:

GGS	Gorlin–Goltz Syndrome
SHH	Sonic HedgeHog
PTCH1	Patched 1
OKC	Odontogenic keratocyst
BCC	Basal cell carcinoma
